# Wogonoside inhibits invasion and migration through suppressing TRAF2/4 expression in breast cancer

**DOI:** 10.1186/s13046-017-0574-5

**Published:** 2017-08-03

**Authors:** Yuyuan Yao, Kai Zhao, Zhou Yu, Haochuan Ren, Li Zhao, Zhiyu Li, Qinglong Guo, Na Lu

**Affiliations:** 1 State Key Laboratory of Natural Medicines, Jiangsu Key Laboratory of Carcinogenesis and Intervention, School of Basic Medicine and Clinical Pharmacy, China Pharmaceutical University, 24 Tongjiaxiang, Nanjing, 210009 People’s Republic of China; 2 Department of Medicinal Chemistry, School of Pharmacy, China Pharmaceutical University, 24 Tongjiaxiang, Nanjing, 210009 People’s Republic of China

**Keywords:** Wogonoside, TNF-α, Twist1, NF-κB, TRAF2, TRAF4

## Abstract

**Background:**

Twist1 is involved in tumor initiation and progression, which especially contributes to tumor invasion and metastasis. Wogonoside is the main in-vivo metabolite of wogonin, and it is also a natural product with potential treatment effects against cancer.

**Methods:**

In this study, we investigated the in-vitro anti-invasion and in-vivo anti-metastasis effects of wogonoside on breast cancer cells and uncovered its underlying mechanism.

**Results:**

The results showed that wogonoside could suppress the growth and metastasis of breast tumor in the orthotopic model of MDA-MB-231 cells. We found that wogonoside could reduce the overexpression of TNF-α, TRAF2 and TRAF4 in later stage of tumor, and improved tumor microenvironment. Therefore, TNF-α was utilized to induce metastases of breast cancer cell in vitro. Wogonoside could inhibit invasion and migration in TNF-α-induced MDA-MB-231, MDA-MB-435, and BT-474 cells. Mechanically, wogonoside inactivated NF-κB signaling through decreasing the protein expression of TRAF2/4, which further inhibited Twist1 expression. Consequently, wogonoside could down-regulate MMP-9, MMP-2, vimentin and CD44v6 expression in TNF-α-induced MDA-MB-231 and MDA-MB-435 cells. Then, these findings were proved in TNF-α + TGF-β1-induced MCF7 cells.

**Conclusions:**

Wogonoside might be a potential therapeutic agent for the treatment of tumor metastasis in breast cancer.

## Background

Breast cancer is a global leading cause of cancer death in women [1–3]. One of the major reasons for such high morbidity and mortality rates of breast cancer is the invasive behavior of breast cancer cells, which leads to cancer metastasis [4]. Several cytokines in the microenvironment could assist breast cancer cells to invade and metastasize. Among these cytokines tumor necrosis factor (TNF-α) is always overexpressed in advanced breast cancer [5].

Twist1 is a bHLH transcription factor that has been known as an essential player in the aggressive phenotype of epithelial-mesenchymal transition (EMT) and cell migration in the developing neural crest [6, 7]. Twist1 is overexpressed in many primary tumors including colon, breast, prostate, and gastric carcinomas [7–9]. In agreement with its role in embryonic cell migration, Twist1 overexpression is associated with the increase in tumor cell migration, invasion, and metastasis [10–12]. Twist1 is also correlated with changes in classical EMT biomarkers such as E-cadherin and vimentin [11, 13]. In addition, downregulation of Twist1 expression suppressed p65-mediated malignancy, which demonstrates that Twist1 is a central modulator downstream from NF-κB [14, 15]. Twist1 promoter contains a functional p65-binding motif, several lines of evidence show that TNF-α-mediated Twist1 expression in breast cancer cells contributes to their aggressive phenotype [15].

Wogonoside is a bioactive flavonoid extracted from the root of *Scutellaria baicalensis Georgi* [16]. It was reported that wogonoside had a preclinical anticancer efficacy in various cancer models, including breast cancer, bladder cancer and hematopoietic malignancies [17]. However, the mechanism of wogonoside inhibiting metastasis remained unclear. In the present study, we evaluated the inhibitory effect of wogonoside in an orthotopic model and TNF-α-induced metastasis. The results showed that wogonoside inhibited growth and metastasis in vivo, and suppressed TNF-α-induced invasion and migration in several breast cancer cells. The mechanism of wogonoside against tumor metastasis was mainly on account of the blocked TRAF2/4-Twist1 axis.

## Methods

### Materials

Wogonoside (>98% purity, Langze Pharmaceutical Co., Ltd., Nanjing, China) was dissolved in dimethylsulfoxide (DMSO) as a stock solution, stored at −20 °C, and diluted with medium before each experiment [17, 18]. MTT [3-(4,5-dimethylthiazol-2-yl)-2,5-diphenyl tetrazolium bromide] was purchased from Sigma Chemical Co. (St. Louis, MO, USA). A nuclear/cytosol fractionation kit (KeyGEN, Nanjing, China) was used according to the manufacturer’s directions. Human recombinant TNF-α and TGF-β1 were from PeproTech Inc. (PeproTech, IL, USA). Primary antibodies against CD44v6 were form Abcam plc. (Abcam, Cambridge, UK), antibodies against E-cadherin, vimentin, p-IκBα and β-Tubulin were from Cell Signaling Technology (CST, MA, USA), antibodies against MMP-9, p-IKKα, IKKα, GAPDH, Lamin A, TNF-α, TRAF2 and TRAF4 were from Bioworld (Bioworld, MN, USA), antibodies against MMP-2, IκBα, NF-κB p65 were from Santa Cruz Biotechnology (Santa Cruz, CA, USA), and antibodies against Twist1 were form Signalway antibody (SAB, MD, USA). IRDye^®^800-conjugated secondary antibodies were from Rockland Inc. (PA, USA).

### Animals

Four-week-old female BALB/c nude mice (Slaccas Shanghai Laboratory Animal Co., Ltd., Shanghai, China) were used for the orthotopic model of MDA-MB-231 cells. The animals were maintained in a pathogen-free environment (23 ± 2 °C, 55 ± 5% humidity) on a 12 h light/12 h dark cycle with food and water supplied *ad* *libitum* throughout the experimental period. Animal study and euthanasia was carried out in strict accordance with the recommendations in the Guide for the Care and Use of Laboratory Animals of the National Institutes of Health. The protocol was approved by the Committee on the Ethics of Animal Experiments of the China Pharmaceutical University.

### Cell culture

Human breast cancer MDA-MB-231 cells, MCF7 cells, MDA-MB-435 cells, BT-474 cells were originally obtained from the Cell Bank of the Shanghai Institute of Cell Biology. The MDA-MB-231 cells, MDA-MB-435 cells and BT-474 cells were cultured in DMEM medium (Gibco, Grand Island, NY) and MCF7 cells were cultured in RPMI 1640 medium (Gibco, Grand Island, NY) both containing 10% fetal bovine serum (Gibco), 100 U/ml penicillin, and 100 μg/ml streptomycin, in a stable environment with 5% CO_2_ at 37 °C.

### Cell viability assay

Cells were plated at a density of 5 × 10^3^ cells/well in 96-well plates. After 24 h culture, the cells were exposed to different concentrations of wogonoside for 48 h in a 5% CO_2_ incubator at 37 °C. Then, MTT was added to the medium and the cells were incubated at 37 °C for 4 h. The supernatant was removed and dimethyl sulfoxide was used to dissolve the precipitate. The absorbance was measured spectrophotometrically at 570 nm.

### Cell attachment assay

The 96-well plates were coated with matrigel (Corning, NY, USA) overnight at 4 °C and blocked with 1% BSA for 4 h at 37 °C. After cells were treated with different concentrations of wogonoside for 48 h, cells were collected in serum-free medium at 5 × 10^5^ cells/ml. Aliquots (100 μl) of the cell suspensions were seeded into the wells and incubated for 1 h at 37 °C. After that, unattached cells were washed thrice with PBS and the attached cells were determined by MTT assay.

### Wound healing assay

Cells were seeded in a six-well plate and allowed to attach overnight, with growth to 80% confluence. The cell monolayers were then wounded with white pipette tips and washed twice with phosphate-buffered saline. The cells were then incubated with wogonoside in medium supplemented with 1% serum for 48 h. The number of migrated cells was determined under an inverted microscopy.

### Cell invasion assay [19]

The transwell chambers (12 mm in diameter, 8 μm pore-size, Millipore, Billerica, MA) were loaded with 0.1 ml of matrigel (Corning, NY, USA) in a 24 well plate at 37 °C for 1 h. After cells were pretreated of wogonoside for 48 h, cells were collected in serum-free medium at a final concentration of 2 × 10^5^ cells/ml. 400 μl cell suspensions were then placed in the upper transwell chamber, and 600 μl medium containing 10% fetal bovine serum was added to the lower compartment. Followed by incubation for 24 h, cells on the upper surface were removed, and invasive cells on the lower surface were fixed with 100% methanol and stained with hematoxylin and eosin. Then quantified by manual counting and three randomly chosen fields were analyzed for each group.

### Western blot

Cells were harvested after pretreatment of wogonoside for 48 h. Western blot was performed as previously described [20]. The membrane was blocked with 5% BSA in PBS at 37 °C for 1 h and incubated overnight at 4 °C with the indicated antibodies, and then with IRDye^®^800-conjugated secondary antibody for 1 h at 37 °C. The samples were visualized with the Odyssey Infrared Imaging System (LI-COR Inc., Lincoln, NE, USA).

### Real-time PCR analysis

Cells were pre-treated with wogonoside for 48 h. The mRNA levels of E-cadherin, vimentin, MMP-9 and Twist1 were then determined with a method described previously [20]. The primer sets used for the PCR amplifications were as follows: *Twist1* (forward, 5′- GGAGTCCGCAGTCTTACGAG-3′, reverse, 5′- TCTGGAGGACCTGGTAGAGG-3′), *E-cadherin* (forward, 5′-CCACCAAAGTCACGCTGAAT-3′, reverse, 5′-GGAGTTGGGAAATGTGAGC-3′), *MMP-9* (forward: 5′-GCAGAGGAATACCTGTACCGC-3′, reverse, 5′-AGGTTTGGAATCTGCCCAGGT-3′), *vimentin* (forward, 5′-ATGAAGGTGCTGCAAAAC-3′, reverse, 5′-GTGACTGCACCTGTCTCCGGTA-3′), *human GAPDH* (forward, 5′-TGGGTGTGAACCATGAGAAG-3′, reverse, 5′-GCTAAGCAGTTGGTGGTGC-3′).

### Transient transfection

Cells were seeded in six-well plates at 70% confluency. The transient transfection assay was performed by using Lipofectamine^®^2000 Transfection Reagent (Thermo, MA, USA) according to the manufacturer’s protocol. Briefly, 8 μl Transfection Reagent and the Twist plasmids (1 μg, Addgene), TRAF2 plasmids (1 μg, Addgene), TRAF4 plasmids (1 μg, Addgene) were respectively diluted in 250 μl medium gently. Then mix the above two diluted medium gently and incubate for 20 min at room temperature. Finally, the complexes were added to each well containing cells and media. The plate was rocked back and forth and incubated at 37 °C in a CO_2_ incubator for 12 h.

### Cytokines detected by ELISA

The concentration of cytokines in supernatant was detected by ELISA kit (Boster Biotechnology, Wuhan, China). Briefly, the supernatant diluted with sample diluent buffer was added to the microwell (100 μl/well) and the plates were then stored in incubator at 37 °C for 90 min. The supernatant was discarded and antibody diluted with antibody diluent buffer was added to the microwell (100 μl/well) and stored in incubator at 37 °C for 1 h. Then plates were washed with PBS for 3 times and Avidin-Biotin-Peroxidase-Complex (ABC) diluted with ABC diluent buffer was added to the microwell. After incubated at 37 °C for 30 min, ABC was discarded and plates were washed with PBS for 5 times. Add the TMB color developing agent to the microwell (90 μl/well) at 37 °C for 20 min and then add the TMB stop solution (100 μl/well). The absorbance was measured at 450 nm.

### Orthotopic model of MDA-MB-231 cells

Orthotopic injections were performed following the previous study with minor modifications [21]. MDA-MB-231 cells (1 × 10^5^/25 μl) were mixed with 25 μl matrigel on ice, and then the cell suspension was quickly injected into the fourth Mammary Fat Pad (MFP). Animal was observed for 30 min until fully recovery. Seventy-seven days later, the mice were randomly divided into three groups (8 mice/group): the negative group (intraperitoneal injection of 0.9% normal saline); the wogonoside-treated group (gavage of 80 mg/kg wogonoside at a frequency of once every other day); and the gemcitabine-positive group (intraperitoneal injection of 80 mg/kg gemcitabine at a frequency of one time every 2 day). Ninety-eight days later, the nude mice were killed and the tumor xenografts were segregated and measured. Additionally, to the blank control group (16 mice, without any drug administration), 8 mice were killed when they lived to 63-day and 98-day respectively, then the tumor xenografts were segregated and measured. Visceral tissue resected from control and test mice were fixed in formalin and tested with H&E staining. Tumor volume (TV) was calculated using the following formula:

$$ TV\ \left({\mathrm{mm}}^3\right)=\frac{D}{2}\times {d}^2 $$

*D* and *d* are the longest and the shortest diameters, respectively. At the same time the animals were weighed twice per week and monitored for mortality throughout the experimental period.

Relative tumor volume (RTV) was calculated according to the equation:

$$ RTV=\frac{V_t}{V_0} $$

*V*_*0*_ is the tumor volume at day 0 and *V*_*t*_ is the tumor volume at day t. And the evaluation index for inhibition was of relative tumor growth ratio

$$ \frac{T}{C}=\frac{T_{RTV}}{C_{RTV}}\times 100\% $$

*T*_*RTV*_ and *C*_*RTV*_ represented RTV of treated and control groups, respectively.

This study was approved in SPF Animal Laboratory of China Pharmaceutical University. In all experiments, the ethics guidelines for investigations in conscious animals were followed, with approval from the local Ethics Committee for Animal Research.

### Immunohistochemistry

The expression of Twist1, E-cadherin, vimentin, MMP-9, TNF-α, TRAF2, and TRAF4 in nude mice model was assessed to the method described previously [22], using a goat-anti-rabbit antibody and an Ultra-Sensitive TMSAP kit. All reagents used in the experiments were supplied by Maixin-Bio Co., Fuzhou, China.

### Statistical analysis

The data were obtained from at least three independent experiments and all data in different experimental groups were expressed as the mean ± SD. We compared TNF-α-treated group or TNF-α + TGF-β1-treated group to control group in vitro*,* and the saline-treated group to control group in vivo. Differences between groups were tested with One-Way ANOVA analysis of variance and Dunnett’s *post* *hoc* test. The changes in tumor weight and tumor volume over time were tested using a random effects mixed model. Metastasis incidence rates were evaluated using percentages of animals withmetastases, and tested using Fisher’s exact test. The significance of differences is indicated at *p < 0.05 and **p < 0.01.

## Results

### Wogonoside suppresses breast cancer growth and metastasis of MDA-MB-231 cells in vivo

**Fig. 1:**
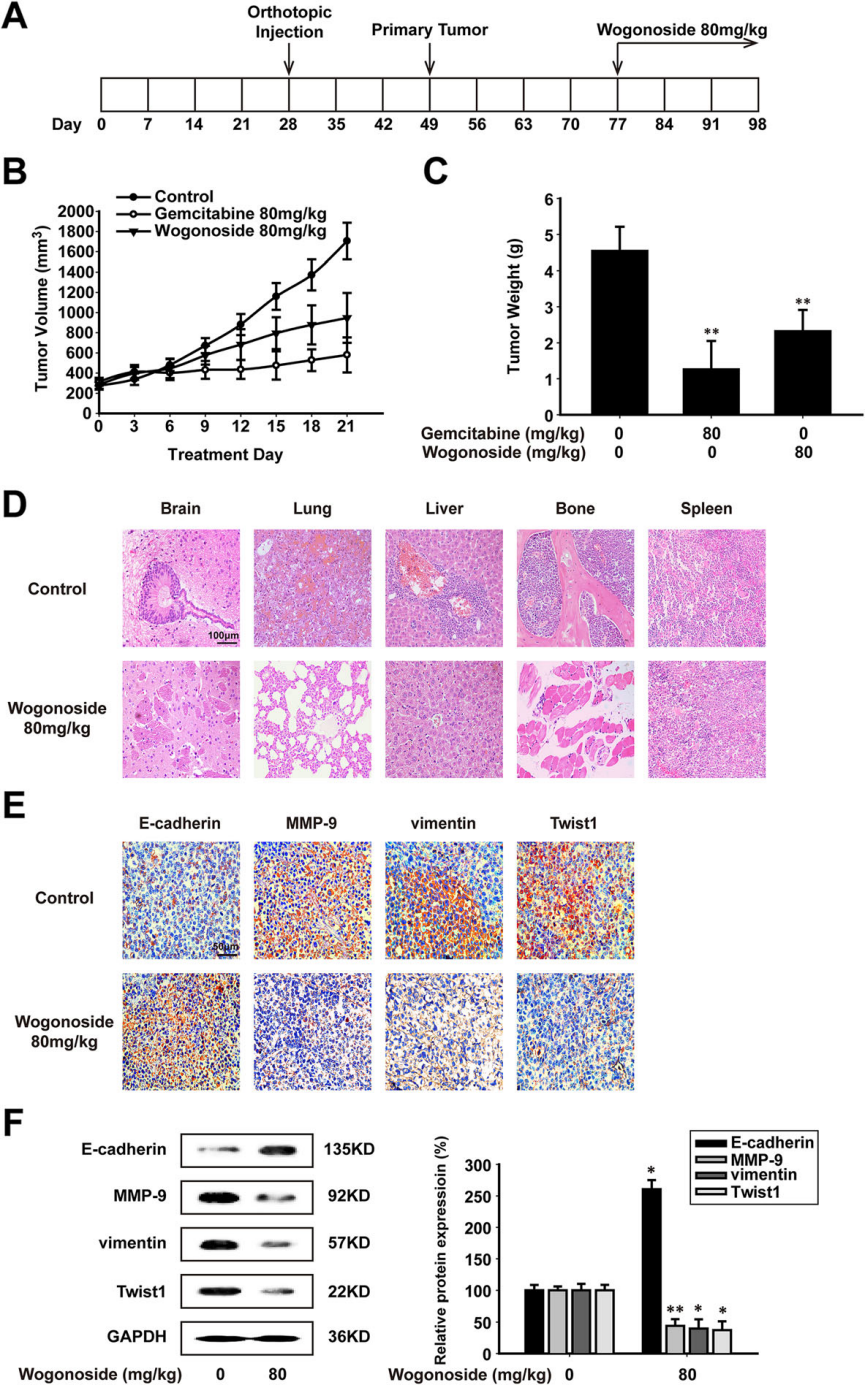
Wogonoside suppresses breast cancer growth and metastasis of MDA-MB-231 cells in vivo*.* **a** Diagram shows the experimental course of MDA-MB-231 orthotopic model. **b** Effect of wogonoside (80 mg/kg) and gemcitabine (80 mg/kg) on tumor growth was investigated in the model of MDA-MB-231 orthotopic model. **c** Effect of wogonoside (80 mg/kg) and gemcitabine (80 mg/kg) on tumor weight was investigated in the model of MDA-MB-231 orthotopic model. **d** H&E stained brains, lungs, livers, bones and spleens of mice from wogonoside-treated and control group to confirm the presence of micrometastases (image magnification: 200×). **e** Immunohistochemical detection of E-cadherin, MMP-9, vimentin and Twist1 protein levels in MDA-MB-231 orthotopic site (image magnification: 400×). **f** The expression of E-cadherin, MMP-9, vimentin and Twist1 proteins were analyzed in MDA-MB-231 orthotopic site by western blot using specific antibodies. A GAPDH antibody was used to check equivalent protein loading. Each experiment was performed at least three times. Data are presented as mean ± SD. *p < 0.05 compared with the control group; **p < 0.01 compared with the control group

The anti-metastatic effect of wogonoside was assessed with orthotopic model of MDA-MB-231 cells in vivo (Fig. 1a). As it is shown in Fig. 1b, during the 21-day treatment, tumor volume was reduced by wogonoside or gemcitabine (80 mg/kg), which showed inhibitory effects on tumor growth of MDA-MB-231 cells. The tumor weight was also decreased compared with the control group (Fig. 1c). The inhibitory rate of wogonoside was about 46%, while that of gemcitabine was approximately 71%. However, as shown in Table 1, eight (100%), three (37.5%) and four (50%) mice were found of metastasis after pathological examination in the control, gemcitabine-treated and wogonoside-treated group respectively. The probability of metastasis was down-regulated by wogonoside (50%), while that of gemcitabine was 62.5%. In the orthotopic model of MDA-MB-231 cells, cancer cells could metastasize to several organs, including brain, lung, liver and bone. Afterwards, we analyzed the incidence of metastasis in each organ according to the pathological section. The results showed that wogonoside could suppress the formation of metastases in brain, lung, liver and bone (Fig. 1d). The instance of organ metastasis was then added up to indicate the risk of metastases in each organ. For example, breast-to-lung cancer metastases were found in 8 mice (100%) in the control group, and lung metastases were found in 4 animals (50%) in the wogonoside-treated group [23] (Table 2). In the primary tumor, we tested the expression of metastasis-associated proteins with immunohistochemistry and western blot assay. It demonstrated that wogonoside could increase the expression of E-cadherin and decrease the expression of MMP-9, vimentin and Twist1 (Fig. 1e and f).

**Table 1:** Probability of metastasis

Treatment	Metastasis incidence(animals with metastases, %)
Control	8/8 (100)
Gemcitabine (80 mg/kg)	3/8 (37.5)^a^
Wogonoside (80 mg/kg)	4/8 (50)^b^

Metastasis incidence is summarized using percentage of animals with metastases and compared between control group, gemcitabine-treated group and wogonoside-treated group using Fisher’s exact test; ^a^ *p* = 0.0256, ^b^ *p* = 0.0769

**Table 2:** Instance of organ metastasis

Treatment	Animals with brain metastasis (%)	Animals with lung metastasis (%)	Animals with liver metastasis (%)	Animals with bone metastasis (%)
Control	3/8 (37.5)	8/8 (100)	6/8 (75)	4/8 (50)
Gemcitabine (80 mg/kg)	1/8 (12.5)^a^	3/8 (37.5)^c^	3/8 (37.5)^e^	2/8 (25)^g^
Wogonoside (80 mg/kg)	1/8 (12.5)^b^	4/8 (50)^d^	3/8 (37.5)^f^	1/8 (12.5)^h^

Metastasis incidence is summarized using percentage of animals with brain, lung, liver and bone metastasis and compared between control group, gemcitabine-treated group and wogonoside-treated group using Fisher’s exact test; ^a^ *p* = 0.5692, ^b^ *p* = 0.5692, ^c^ *p* = 0.0256, ^d^ *p* = 0.0769, ^e^ *p* = 0.3174, ^f^ *p* = 0.3174, ^g^ *p* = 0.6084, ^h^ *p*. = 0.2821

### TNF-α and TRAF2/4 were overexpressed in late stage of metastatic breast cancer

**Fig. 2:**
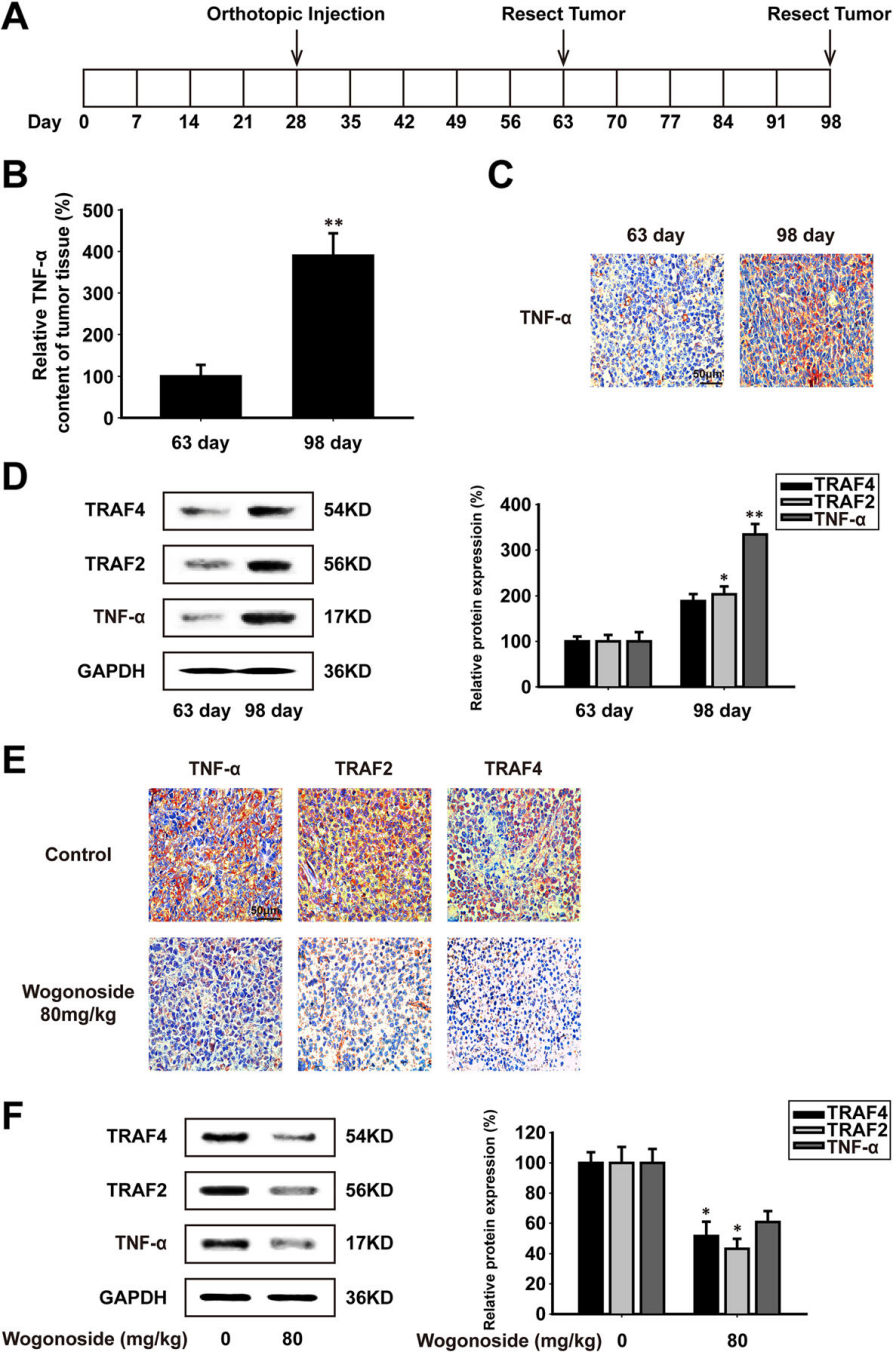
TNF-α and TRAF2/4 were overexpressed in late stage of metastatic breast cancer. **a** Diagram shows the experimental course of MDA-MB-231 orthotopic model (*n* = 8). **b** The effect of wogonoside on TNF-α content of tumor tissue. **c** Immunohistochemical detection of TNF-α protein levels in 63-day and 98-day MDA-MB-231 orthotopic site (image magnification: 400×). **d** The expression of TRAF4, TRAF2 and TNF-α proteins were analyzed in 63-day and 98-day MDA-MB-231 orthotopic site by western blot using specific antibodies. A GAPDH antibody was used to check equivalent protein loading. **e** Immunohistochemical detection of TRAF4, TRAF2 and TNF-α protein levels in MDA-MB-231 orthotopic site (image magnification: 400×). **f** The expression of TRAF4, TRAF2 and TNF-α proteins were analyzed in MDA-MB-231 orthotopic site by western blot using specific antibodies. Each experiment was performed at least three times. Data are presented as mean ± SD. *p < 0.05 compared with the control group; **p < 0.01 compared with the control group

Mice tumors resected at different time periods were shown in Fig. 2a. By Elisa assay and immunohistochemical analysis, we found that the content of TNF-α in 98-day primary tumors was increased, compared to 63-day primary tumors (Fig. 2b and c). Meanwhile, the expressions of TNF-α, TRAF2 and TRAF4 were also enhanced in 98-day primary tumors (Fig. 2d). Coincidentally, as shown in Fig. 2e and f, the immunohistochemistry assay and western blot assay verified that wogonoside could reduce the protein expression of TNF-α, TRAF2, and TRAF4. Therefore, we wondered that whether wogonoside inhibited metastasis through suppressingTRAF2/4 expression.

### Wogonoside inhibits TNF-α-induced migration, adhesion and invasion in MDA-MB-231, MDA-MB-435, and BT-474 cells

**Fig. 3:**
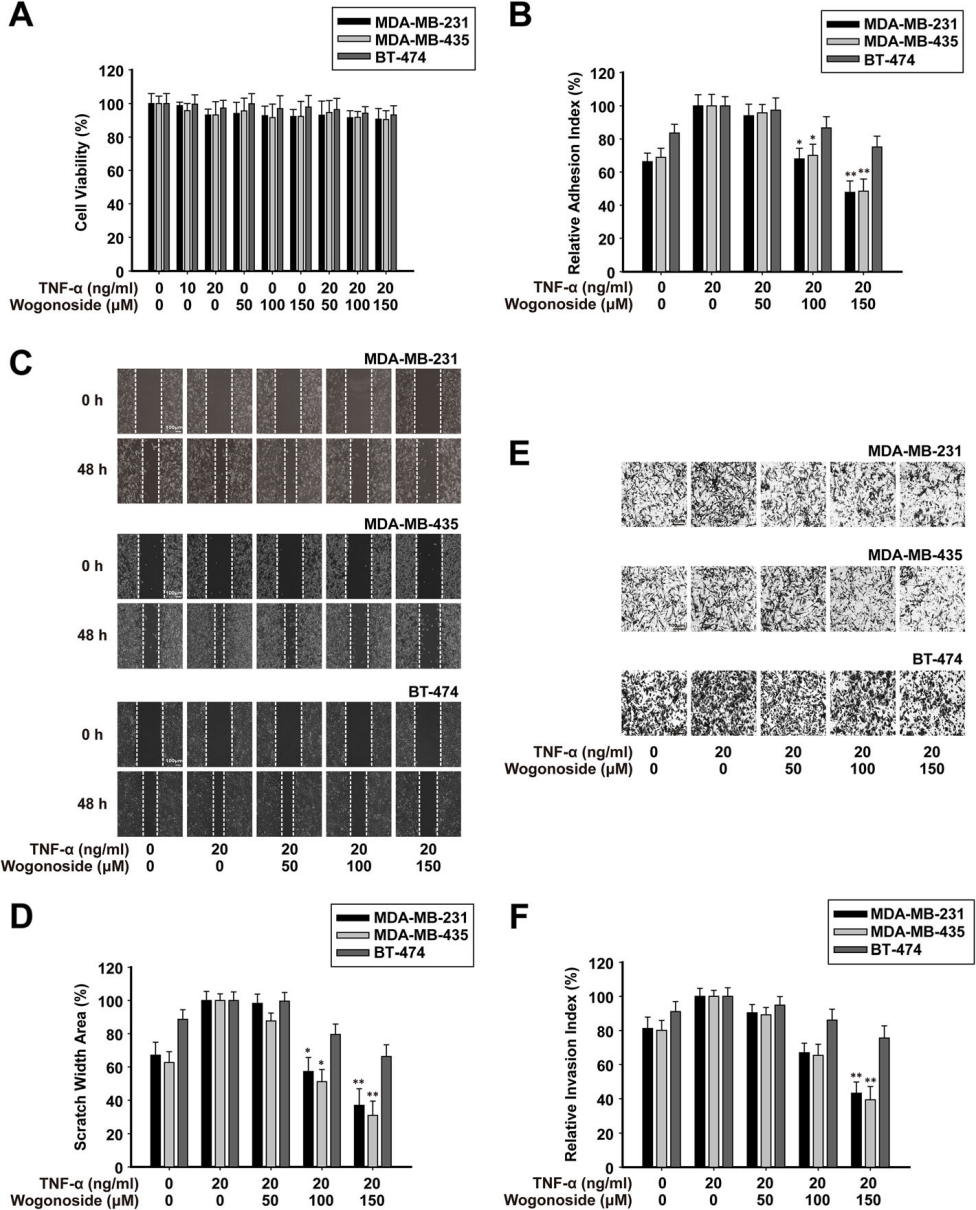
Wogonoside inhibits TNF-α-induced migration, adhesion and invasion in MDA-MB-231, MDA-MB-435, and BT-474 cells. MDA-MB-231, MDA-MB-435, and BT-474 cells were exposed to different concentrations of TNF-α and wogonoside for 48 h. **a** Effect of wogonoside on cell viability by MTT assay. **b** 100 μl cell suspension (2 × 10^5^ cells/ml) was added to the 96 wells which are pre-coated with matrigel. After incubating for 60 min, adherent cells were determined by MTT assay. **c-d** A monolayer of cells was scraped with a pipette tip and then treated with TNF-α and wogonoside. The migrating cells were assessed with a microscope equipped with a camera (image magnification: 100×). **e**-**f** The invasive ability was evaluated by a matrigel-coated transwell invasion assay (image magnification: 200×). Each experiment was performed at least three times. Data are presented as mean ± SD. *p < 0.05 compared with the control group; **p < 0.01 compared with the control group

As shown in Fig. 3a, the result of MTT assay revealed that the treatment of TNF-α (20 ng/ml) and wogonoside (0, 50, 100 and 150 μM) caused no significant cytotoxicity in MDA-MB-231, MDA-MB-435, and BT-474 cells. These concentrations were then applied to all subsequent experiments. Cancer cell adhesion to basement membranes is important for tumor invasion since it is a key step in proteinase-dependent cell locomotion. The results of cell attachment assay showed that the adhesive capabilities of TNF-α-induced MDA-MB-231, MDA-MB-435, and BT-474 cells were decreased after treatment of wogonoside (Fig. 3b).

We next examined the effect of wogonoside on the TNF-α-induced migration of these three cells. As shown in Fig. 3c and d, migrated cells were quantified by scratch width. The inhibitory rate of wogonoside (50, 100 and 150 μM) was about 1%, 43% and 63% in MDA-MB-231 cells, 12%, 49% and 69% in MDA-MB-435 cells, 1%, 20% and 34% in BT-474 cells, respectively.

Then, we investigated the effects of wogonoside on the TNF-α-induced invasion of MDA-MB-231, MDA-MB-435, and BT-474 cells in vitro. We found that cells in the control group were able to invade freely through the matrigel, whereas this ability was inhibited by wogonoside. As shown in Fig. 3e and f, wogonoside could inhibit the invasion of MDA-MB-231, MDA-MB-435, and BT-474 cells in a concentration-dependent manner, and the inhibition rate at 150 μM was about 57% in MDA-MB-231 cells, 60% in MDA-MB-435 cells, and 24% in BT-474 cells.

### Wogonoside inhibits the expression of metastasis-associated proteins through suppressing Twist1 expressing in breast cancer cells

**Fig. 4:**
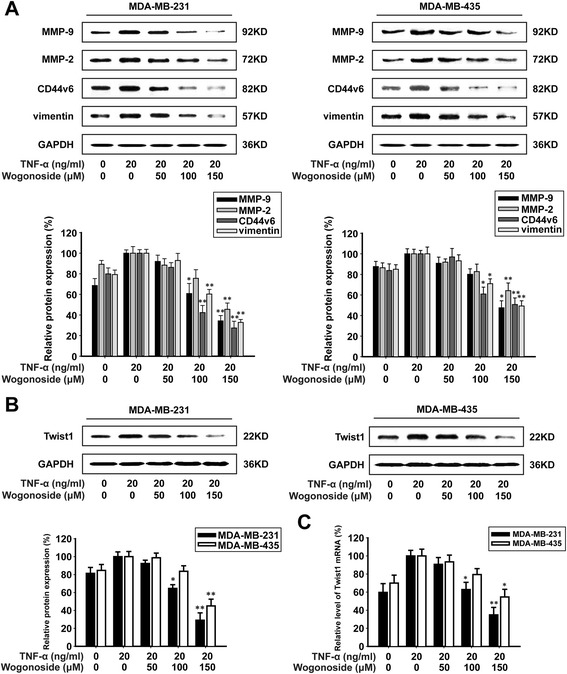
Wogonoside inhibits the expression of metastasis-associated proteins through suppressing Twist1 expressing in breast cancer cells. MDA-MB-231 and MDA-MB-435 cells were exposed to different concentrations of TNF-α and wogonoside for 48 h. **a** The expression of MMP-9, MMP-2, CD44v6, and vimentin proteins in the cells were analyzed by western blot using specific antibodies. **b** The expression of Twist1 protein in the cells was analyzed by western blot using specific antibodies. **c** Twist1 mRNAs were measured with real-time PCR. GAPDH was used as the internal control. The relative levels were calculated as the ratio of the relative biomarker mRNA to GAPDH mRNA. Each experiment was performed at least three times. Data are presented as mean ± SD. *p < 0.05 compared with the control group; **p < 0.01 compared with the control group

In the process of cancer metastasis, MMP-9, CD44v6 and vimentin are responsible for cell migration, invasion and cell-matrix adhesion. Therefore, the effects of wogonoside on the expression of MMP-9, MMP-2, CD44v6, vimentin and Twist1 in MDA-MB-231 and MDA-MB-435 cells were detected by western blot analysis. As shown in Fig. 4a, we observed that wogonoside reduced the expression of MMP-9, MMP-2, CD44v6 and vimentin in TNF-α-induced MDA-MB-231 and MDA-MB-435 cells. TNF-α could induce Twist1 expression and overexpression of Twist1 could promote further metastasis, which increased the expression of MMP-9, MMP-2, CD44v6 and vimentin in cancer cells. Therefore, we detected Twist1 expression. As shown in Fig. 4b and c, wogonoside decreased the protein and mRNA expression of Twist1, which suggested that wogonoside could inhibit Twist1 expression at the transcriptional level. The inhibition rate of Twist1 mRNA level was about 9%, 37% and 65% in TNF-α-induced MDA-MB-231 cells and 7%, 21% and 45% in TNF-α-induced MDA-MB-435 cells.

### Wogonoside inhibits TNF-α-induced NF-κB signaling through suppressing the expression of TRAF2/4

**Fig. 5:**
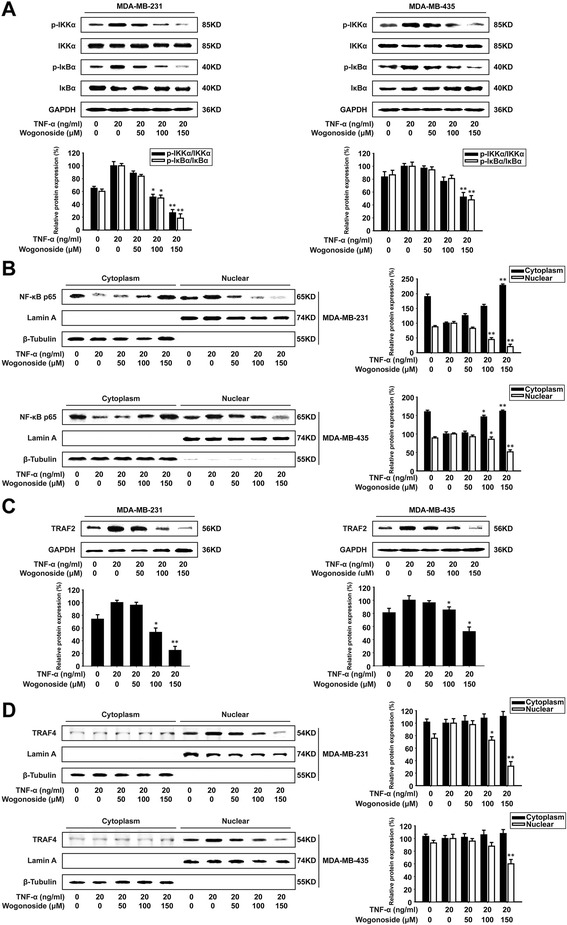
Wogonoside inhibits TNF-α-induced NF-κB signaling through suppressing the expression of TRAF2/4. MDA-MB-231 and MDA-MB-435 cells were exposed to different concentrations of TNF-α and wogonoside for 48 h. **a** The expression of p-IKKα, IKKα, p-IκBα and IκBα proteins in the cells were analyzed by western blot using specific antibodies. **b** The expression and localization of NF-κB p65 protein in the cells was analyzed by western blot using specific antibodies. **c** The expression of TRAF2 protein in the cells was analyzed by western blot using specific antibodies. **d** The expression and localization of TRAF4 protein in the cells was analyzed by western blot using specific antibodies. Each experiment was performed at least three times. Data are presented as mean ± SD. *p < 0.05 compared with the control group; **p < 0.01 compared with the control group

Previous study has demonstrated that NF-κB pathway is involved with the regulation of Twist1 expression at the level of transcription. Therefore, we examined the key kinases in NF-κB signaling and the nuclear translocation of p65. The results showed that wogonoside inhibited the phosphorylation of IκBα and IKKα in TNF-α-induced MDA-MB-231 and MDA-MB-435 cells (Fig. 5a). And the nuclear translocation of NF-κB p65 was also inhibited by wogonoside in TNF-α-induced MDA-MB-231 and MDA-MB-435 cells (Fig. 5b).

Since TRAF2 complex is the upstream of NF-κB signaling, which could be responsible for the phosphorylation of IKK, we detected the inhibitory effect of wogonoside on TRAF2 expression. The results showed that wogonoside could inhibit TRAF2 expression in TNF-α-induced MDA-MB-231 and MDA-MB-435 cells (Fig. 5c). In addition, according to the experiment of orthotopic model, wogonoside could decrease TRAF2 and TRAF4 in vivo. Therefore, we further investigated whether TRAF4 expression was also decreased in these two cells. We found that wogonoside mainly inhibited the nuclear protein level of TRAF4 instead of the cytoplasmic protein level (Fig. 5d).

### Wogonoside inhibits TNF-α + TGF-β1-induced migration, adhesion and invasion in MCF7 cells in vitro

**Fig. 6:**
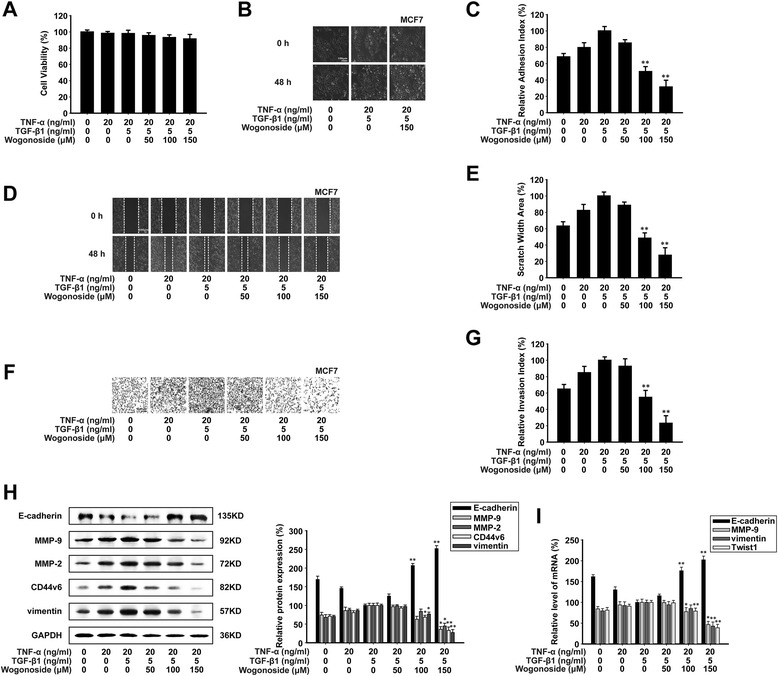
Wogonoside inhibits TNF-α + TGF-β1-induced migration, adhesion and invasion in MCF7 cells in vitro*.* MCF7 cells were exposed to different concentrations of TNF-α, TGF-β1 and wogonoside for 48 h. **a** An MTT assay showed that TNF-α, TGF-β1 and wogonoside had no effect on cell viability. **b** Morphological changes in MCF7 cells were observed under an inverted lightmicroscope (400×). **c** 100 μl cell suspension (2 × 10^5^ cells/ml) was added to the 96 wells which are pre-coated with matrigel. After incubating for 60 min, adherent cells were determined by MTT assay. **d**-**e** A monolayer of cells was scraped with a pipette tip and then treated with TNF-α, TGF-β1 and wogonoside. The migrating cells were assessed with a microscope equipped with a camera (image magnification: 100×). **f**-**g** The invasive ability was evaluated by a matrigel-coated transwell invasion assay (image magnification: 200×). **h** The expression of E-cadherin, MMP-9, MMP-2, CD44v6, and vimentin proteins in the cells were analyzed by western blot using specific antibodies. **i** E-cadherin, MMP-9, vimentin and Twist1 mRNAs were measured with real-time PCR. GAPDH was used as the internal control. The relative levels were calculated as the ratio of the relative biomarker mRNA to GAPDH mRNA. Each experiment was performed at least three times. Data are presented as mean ± SD. *p < 0.05 compared with the control group; **p < 0.01 compared with the control group

Since the treatment of TNF-α alone in MCF7 cells could not induce EMT efficiently, we added TGF-β1 (5 ng/ml) to promote the EMT process. An MTT assay showed that TNF-α (20 ng/ml) and TGF-β1 (5 ng/ml) or the combination of wogonoside, TNF-α and TGF-β1 had no cytotoxic effect (Fig. 6a). These concentrations were used in the following experiments. As shown in Fig. 6b, we found that MCF7 cells transformed from an epithelial morphology to an elongated fibroblast-like cell morphology under the treatment of TNF-α and TGF-β1. However, wogonoside (150 μM) could inhibit TNF-α + TGF-β1-induced morphological changes in MCF7 cells. The results of cell attachment assay showed that the adhesive capabilities of TNF-α + TGF-β1-induced MCF7 cells were decreased after treatment of wogonoside (Fig. 6c). Afterwards, we investigated the anti-invasive and anti-migratory effects of wogonoside on TNF-α + TGF-β1-triggered EMT with matrigel invasion and wound healing assays. Wogonoside inhibited the migration of TNF-α + TGF-β1-stimulated MCF7 cells across the wounded space in a concentration-dependent manner (Fig. 6d and e). And treatment with wogonoside could also reduce the invasiveness of TNF-α + TGF-β1-induced MCF7 cells through matrigel (Fig. 6f and g).

As TNF-α and TGF-β1 triggered EMT process in MCF7 cells, we investigated the effect of wogonoside on EMT biomarkers. Quantization of western blot assay showed that wogonoside up-regulated the protein and mRNA expression of E-cadherin while it down-regulated the protein and mRNA expression of MMP-9 and vimentin in a concentration-dependent manner (Fig. 6h and i).

### Wogonoside inhibits Twist1 expression through suppressing TRAF2/4 expression in MCF7 cells

**Fig. 7:**
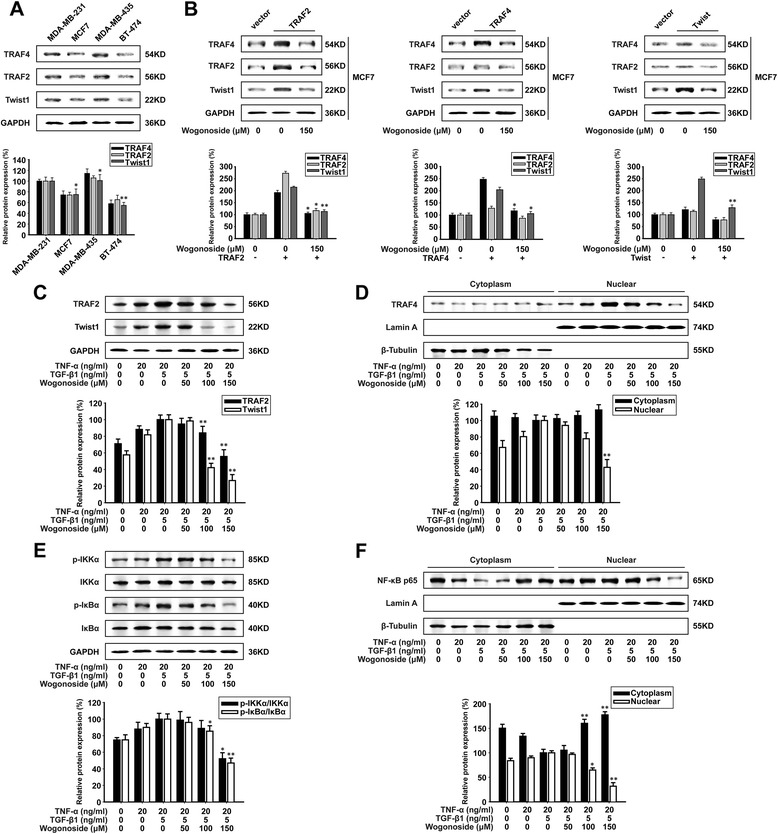
Wogonoside inhibits Twist1 expression through suppressing TRAF2/4 expression in MCF7 cells. MCF7 cells were exposed to different concentrations of TNF-α, TGF-β1 and wogonoside for 48 h. **a** TRAF4, TRAF2 and Twist1 expression in MDA-MB-231, MCF7, MDA-MB-435, and BT-474 cells were analyzed by western blot using specific antibodies. Comparison was made with the expression in MDA-MB-231 cells. **b** MCF7 cells were transfected with TRAF4, TRAF2 and Twist plasmid. TRAF4, TRAF2 and Twist1 expression with treatment of wogonoside were analyzed by western blot. **c** The expression of TRAF2 and Twist1 protein in the cells were analyzed by western blot using specific antibodies. **d** The expression and localization of TRAF4 protein in the cells was analyzed by western blot using specific antibodies. **e** The expression of p-IKKα, IKKα, p-IκBα and IκBα proteins in the cells were analyzed by western blot using specific antibodies. **f** The expression and localization of NF-κB p65 protein in the cells was analyzed by western blot using specific antibodies. Each experiment was performed at least three times. Data are presented as mean ± SD. *p < 0.05 compared with the control group; **p < 0.01 compared with the control group

As shown in Fig. 7a, the expression of TRAF2, TRAF4, and Twist1 in MDA-MB-231, MCF7, MDA-MB-435, and BT-474 cells were detected by western blot analysis. There were different protein levels of Twist1 in four cells: MDA-MB-231 and MDA-MB-435 with high expression (100%), MCF7 with medium expression (75%), and BT-474 with low expression (55%).

Since MCF7 cells exhibited lower expression of Twist1, TRAF2 and TRAF4, we transfected the overexpressed plasmids of TRAF2, TRAF4 and Twist into MCF7 cells. The results showed that transfection of TRAF2 and TRAF4 plasmids could induce Twist1 expression while transfection of Twist plasmids could induce TRAF2/4 expression. On the other hand, the overexpressed proteins of TRAF2, TRAF4 and Twist1 were all decreased by wogonoside (Fig. 7b).

Indeed, the stimulation of TNF-α and TGF-β1 could also enhance the expression of TRAF2, TRAF4 and Twist1 in MCF7 cells. Hence, we investigated whether the mechanism of wogonoside against EMT was involved with TRAF2/4 expression. The results showed that wogonoside inhibited the total protein of TRAF2 and the nuclear protein level of TRAF4 in TNF-α + TGF-β1-induced MCF7 cells (Fig. 7c and d). Correspondingly, wogonoside could inhibit the phosphorylation of IκBα and IKKα in TNF-α + TGF-β1-induced MCF7 cells (Fig. 7e). Meanwhile, as shown in Fig. 7f, nuclear translocation of NF-κB p65 was also inhibited by wogonoside.

## Discussion

Wogonoside is not only a bioactive component of *Scutellaria baicalensis Georgi*, but also a main in-vivo metabolite of wogonin. Wogonin was reported to exhibit anti-metastatic effects in various solid tumors, such as breast cancer, melanoma and hepatocellular cancer [24–26]. Although the anti-metastatic effect of wogonin was proved, the mechanism of wognin has not been fully revealed and whether its in-vivo main metabolite has the same anti-metastatic effect was not clear. In this study, we demonstrated that wogonoside possessed the anti-metastatic potential in breast cancer and uncovered its mechanism for the first time.

MDA-MB-231 is a breast cancer cell line from patients with Triple-negative breast cancer (TNBC), which is a subtype of breast cancer with poor prognosis and limited treatment options. As the drug of first choice for patients suffered with TNBC, gemcitabine, a pyrimidine analog, could suppress DNA replication and induce apoptosis of breast cancer cells with a great damage to bone marrow, liver and kidney. As an in-vivo metabolite of wogonin, wogonoside showed a lower toxicity and did not act as cytotoxic agent and we took gemcitabine as a positive drug in comparison with the effect of wogonoside in breast cancer. Although the inhibitory rate of wogonoside on the growth of primary tumor was lower than that of gemcitabine, the effect of wogonoside on experimental metastasis was comparable to gemcitabine. It suggested that wogonoside possessed the potential anti-tumor activity in the treatment of metastatic breast cancer. According to our results, wogonoside could suppress tumor metastasis through inhibition of primary tumor invasion by increasing the expression of E-cadherin and decreasing the expression of MMP-9, vimentin and Twist1. On the other hand, we found the expression of TNF-α, TRAF2 and TRAF4 was enhanced in the primary tumor as time goes on. This might be due to the internal tumor necrosis and the pro-inflammatory environment, which promotes overexpression of TNF-α and activation of NF-κB pathway in later stage of tumor. And these factors always promote invasion and metastasis. Hence, TNF-α was used to induce the metastatic process of breast cancer cells*.*

Tumor metastasis accounts for 90% of cancer-associated deaths, in which invasion plays a critical role in metastasis [27]. During invasion, tumor cells firstly lose cell-cell junctions, subsequently degrade, remodel, and adhere to the surrounding ECM and eventually migrate through ECM to the distance sites [28]. Therefore, wound healing assay, transwell invasion assay and cell adhesion assay were used to measure the anti-migration, anti-invasion and anti-adhesion effect of wogonoside in vitro. We found that wogonoside could inhibit invasion and migration in TNF-α-induced MDA-MB-231, MDA-MB-435 and BT-474 cells. Meanwhile, the abnormal expression of MMP-9, MMP-2, vimentin and CD44v6 in cancer cells would lead to decreased adhesion, enhanced migration and invasion. Thus, Wogonoside inhibited metastasis-related protein expression to block TNF-α-induced metastatic process. Twist1 is a master regulator of morphogenesis, which can induce EMT to facilitate breast tumor metastasis [6]. We found that wogonoside reduced the mRNA and protein expression of Twist1 in TNF-α-induced MDA-MB-231 and MDA-MB-435 cells, which indicated that the anti-metastatic effect of wogonoside in breast cancer was dependent of Twist1 expression.

Activation of NF-κB pathway is associated with Twist1 expression and EMT in cancer cells [29, 30]. NF-κB activation in response to inflammatory cytokines and growth factors is frequently observed in metastatic breast cancer cells. NF-κB has been shown to be essential for EMT and metastasis in a model of breast cancer progression [31]. We found wogonoside could inhibit the activation of NF-κB signaling through suppressing the expression of TRAF2 and TRAF4. Upon TNF-α stimulation, TRAF2 is recruited and involved in the activation of the receptor. The receptor complex collaborates with TRAF2 to bring the TGF-β-activated kinase 1 (TAK1) kinase complex close to the IKK complex, which is phosphorylated afterwards [32]. And another important member of TRAF family proteins, TRAF4 is mainly expressed in nucleus. The nuclear expression of TRAF4 is correlated with poor survival in breast cancer patients [33]. TRAF4 is also required for the activation of TAK1 and TGF-β-induced EMT [34]. Therefore, TRAF2 and TRAF4 could both positively regulate the activation of NF-κB pathway, which promoted Twist1 expression transcriptionally. Consequently, wogonoside down-regulated TRAF2 and TRAF4 expression to block NF-κB signaling and Twist1 transcprition.

As Twist1 is a critical regulator in EMT process, we investigated the inhibitory effect of wogonoside on EMT process in the model of TNF-α + TGF-β1-induced MCF7 cells. During EMT, cells lose their epithelial characteristics, including cell adhesion and polarity, and acquire a mesenchymal morphology and the ability to migrate. Biochemically, cells switch off the expression of epithelial markers such as adherens junction protein E-cadherin and turn on mesenchymal markers including vimentin and fibronectin [35]. We demonstrated that wogonoside inhibited migration, invasion and cytoskeletal remodeling in TNF-α + TGF-β1-induced MCF7 cells. In addition, wogonoside increased E-cadherin expression and reduced vimentin expression through decreasing Twist1 expression. Meanwhile, when TNF-α and TGF-β1 activated NF-κB pathway, wogonoside could also suppress the expression of TRAF2 and TRAF4. It further verified that wogonoside had an inhibitory effect on TRAF2/4 expression after exogenous stimulation.

## Conclusions

In conclusion, wogonoside could inhibit TRAF2/4 expression, thus inactivate NF-κB signaling, and finally suppress Twist1 protein content and EMT process. Accordingly, wogonoside inhibited the invasion and migration of breast cancer cells in vitro and in vivo. Therefore, wogonoside might be a potential therapeutic agent for the treatment of metastatic breast cancer.

## Abbreviations

ABC: Avidin-biotin-peroxidase-complex
EMT: Epithelial-mesenchymal transition
MFP: Mammary fat pad
RTV: Relative tumor volume
TAK1: TGF-β-activated kinase 1
TGF-β: Transforming growth factor-β
TNBC: Triple-negative breast cancer
TNF-α: Tumor necrosis factor-α
TV: Tumor volume
