# Correction to: Wogonoside inhibits invasion and migration through suppressing TRAF2/4 expression in breast cancer

**DOI:** 10.1186/s13046-019-1447-x

**Published:** 2019-10-31

**Authors:** Yuyuan Yao, Kai Zhao, Zhou Yu, Haochuan Ren, Li Zhao, Zhiyu Li, Qinglong Guo, Na Lu

**Affiliations:** 1 State Key Laboratory of Natural Medicines, Jiangsu Key Laboratory of Carcinogenesis and Intervention, School of Basic Medicine and Clinical Pharmacy, China Pharmaceutical University, 24 Tongjiaxiang, Nanjing, 210009 People’s Republic of China; 2 Department of Medicinal Chemistry, School of Pharmacy, China Pharmaceutical University, 24 Tongjiaxiang, Nanjing, 210009 People’s Republic of China

**Correction to: J Exp Clin Cancer Res (2017) 36:103**

**https://doi.org/10.1186/s13046-017-0574-5**

In the original publication of this article [1], there are mistakes in Fig. 3c and Fig. 3e.

The corrected Fig. 3 should be:

**Figure 1:**
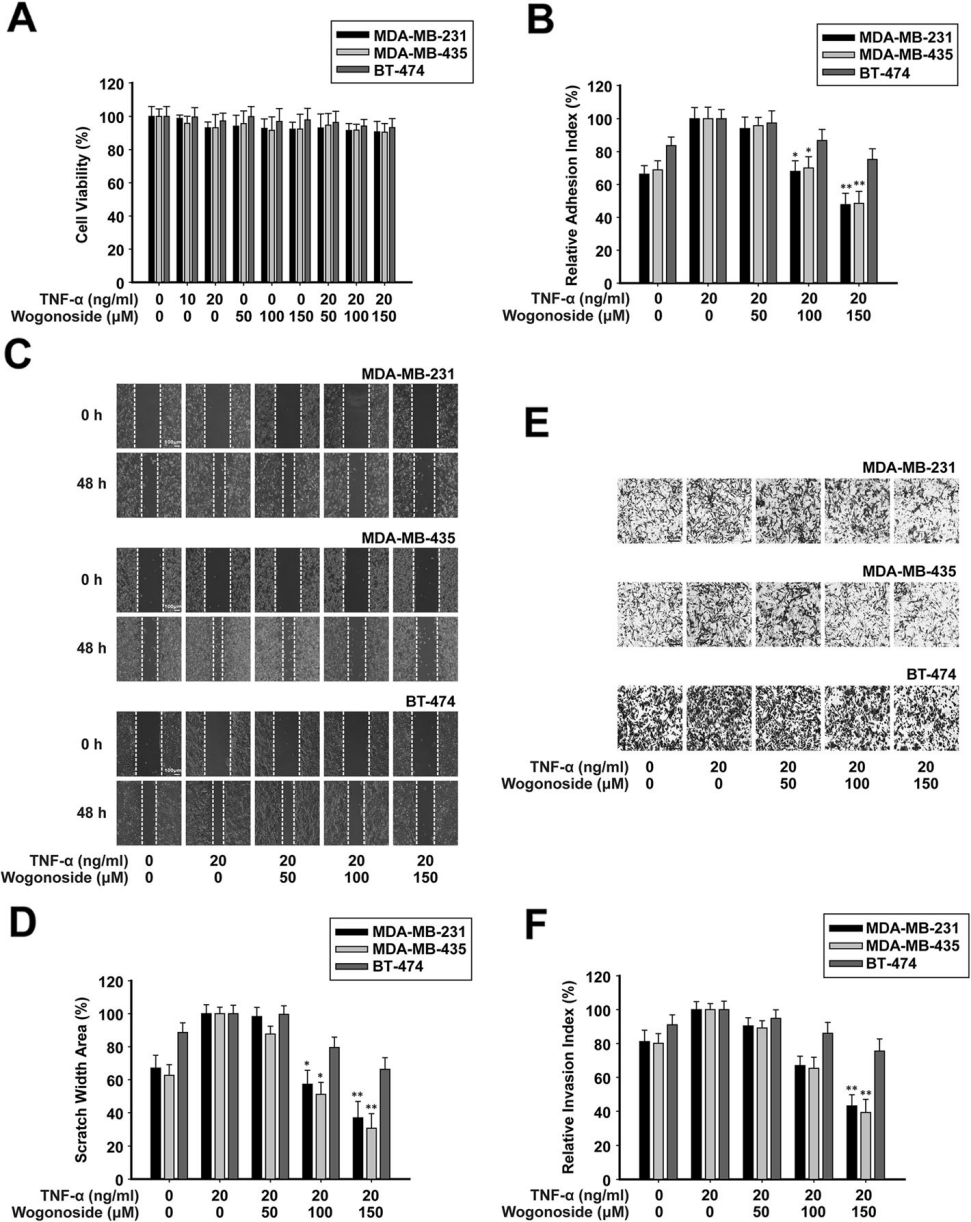
Fig. 3
